# Osseointegrated prostheses for rehabilitation following amputation: The pioneering Swedish model

**DOI:** 10.1007/s00113-017-0331-4

**Published:** 2017-02-22

**Authors:** Yan Li, Rickard Brånemark

**Affiliations:** 1 Center of Orthopedic Osseointegration (COO), Center for Advanced Reconstruction of Extremities (C.A.R.E.), Sahlgrenska University Hospital, Department of Orthopedics, Gothenburg University, Gothenburg, Sweden; 2 Division of Orthopedics and Biotechnology, Department of Clinical Intervention and Technology (CLINTEC), Karolinska Institutet, Stockholm, Sweden; 3 Department of Orthopedics, Sahlgrenska Academy, Gothenburg University, Gothenburg, Sweden; 4 International Center for Osseointegration Research, Education and Surgery (iCORES), Department of Orthopedics, University of California, San Francis, USA

**Keywords:** Osseointegration Artificial limbs, Amputation, Prosthesis implantation, OPRA, Prostheses and implants, Künstliche Gliedmaßen, Amputation, Prothesenimplantation, OPRA, Prothesen und Implantate

## Abstract

The direct attachment of osseointegrated (OI) prostheses to the skeleton avoids the inherent problems of socket suspension. It also provides physiological weight bearing, improved range of motion in the proximal joint, as well as osseoperceptive sensory feedback, enabling better control of the artificial limbs by amputees. The present article briefly reviews the pioneering efforts on extremity osseointegration surgeries in Sweden and the development of the OPRA (Osseointegrated Prostheses for the Rehabilitation of Amputees) program. The standard implant design of the OPRA system and surgical techniques are described as well as the special rehabilitation protocols based on surgical sites. The results of long-term follow-up for transradial, transhumeral, and thumb amputee operations are briefly reported including the prospective study of transfemoral amputees according to OPRA protocol. The importance of refinement on implant designs and surgical techniques based on the biomechanical analysis and early clinical trials is emphasized. Future aspects on osseointegration surgery are briefly described, including novel treatment options using implanted electrodes.

## Zusammenfassung

Durch die direkte Anbringung osseointegrierter (OI-)Prothesen an das Skelett können die Probleme der Schaftprothesenversorgung vermieden werden. Mit der OI-Prothese lassen sich eine physiologische Gewichtsbelastung, ein verbesserter Bewegungsumfang des proximalen Gelenks und eine osseoperzeptive sensorische Rückkopplung erzielen, was eine bessere Steuerung künstlicher Gelenke durch Patienten mit Amputationen ermöglicht. Im vorliegenden Beitrag wird eine kurze Übersicht über die wegweisenden Leistungen in der Osseointegrationschirurgie der Extremitäten in Schweden und über die Entwicklung des OPRA-Programms (Osseointegrated Prostheses for the Rehabilitation of Amputees) gegeben. Dabei werden das Standardimplantatdesign im OPRA-System und chirurgische Techniken sowie spezielle Rehabilitationsschemata je nach Operationsgebiet dargestellt. Über die Ergebnisse der Langzeitnachbeobachtung nach Operation von Patienten mit transradialer, transhumeraler und Daumenamputation einschließlich der prospektiven Studie an Patienten mit transfemoraler Amputation gemäß OPRA-Protokoll wird kurz berichtet. Die Bedeutung von Verbesserungen des Implantatdesigns und chirurgischer Techniken auf der Basis biomechanischer Analyse und früher klinischer Studien wird hervorgehoben. Zukünftige Aspekte der Osseointegrationschirurgie einschließlich neuer Therapieoptionen unter Verwendung von implantierten Elektroden werden kurz beschrieben.

## Background

Despite the improvements in medical and surgical interventions for limb salvage procedures, the amputation numbers remain high in the world due to the aging population, civilian accidents, local wars, and terrorism attacks [1, 2]. Prostheses are aimed to enhance mobility, independence, safety, and quality of life for amputees [3]. Evidence of prosthesis usage can be dated back to ancient Egyptians. The earliest documented functional lower-limb prosthesis was unearthed in Italy, probably from 300 B.C. The weight-bearing part of the prosthesis was made of bronze and iron, combined with a wooden/leather socket for connecting the residual limb [4]. The material and technique evolved over centuries, while the socket remains as a critical part for prosthesis connection. The socket design, however, frequently places the residual limb under excessive stresses and pistoning (vertical movements within the socket) and results in skin irritation and ulcers, which are often regarded as the major reasons for prosthesis rejection by amputees [5].

The direct attachment of an osseointegrated (OI) prosthesis to the skeleton avoids the inherent problems of socket suspension. It also provides physiological weight bearing, improved range of motion in the proximal joint, and osseoperceptive sensory feedback, thus, enabling better control of the artificial limbs by amputees [6–8]. The cumulative success rate of 92% at the 2‑year follow-up [9] and reports on the dramatic improvements of quality of life for transfemoral amputees [6, 9–11] led to the approval of the Osseoanchored Prostheses for the Rehabilitation of Amputees (OPRA) device recently by the Food and Drug Administration (FDA) for rehabilitation of above-the-knee amputees. The present article briefly reviewed the pioneering efforts on extremity osseointegration surgeries in Sweden and the development of the OPRA program.

## Historical aspects

In the early 1960s, the Swedish researcher Per-Ingvar Brånemark discovered in his microcirculation studies that his titanium chambers were firmly maintained in rabbit tibia without severe soft tissue reaction or loosening. When the experiments were finished, the titanium chambers could not be removed from the bone. These unexpected findings gave P.-I. Brånemark the idea that titanium implants could be used as a restoration option for tooth loss. After successful animal experiments with intraosseous anchorage of dental prostheses [12], P.-I. Brånemark completed the first human trial in an edentulous patient in 1965. The long-term success of a series of clinical trials confirmed the advantage of the functional and structural connection between living bone and the titanium implant, which he later named “osseointegration” [13].

The application of osseointegration for amputee rehabilitation started in 1990s, mainly based on the dental and craniofacial osseointegration experience and the biomechanical studies of P.-I. Brånemark’s son, Rickard Brånemark, who later became the Chief Surgeon and Director of the Center for Orthopedic Osseointegration (COO) at Sahlgrenska University Hospital in Gothenburg, Sweden. R. Brånemark and coworkers evaluated the biomechanics of bone-anchored implants during healing, after irradiation, in experimental arthritis, in rat, rabbit, dog, and human [14]. This experimental work became the basis for implant designs and rehabilitation protocols for extremity osseointegrations.

**Fig. 1:**
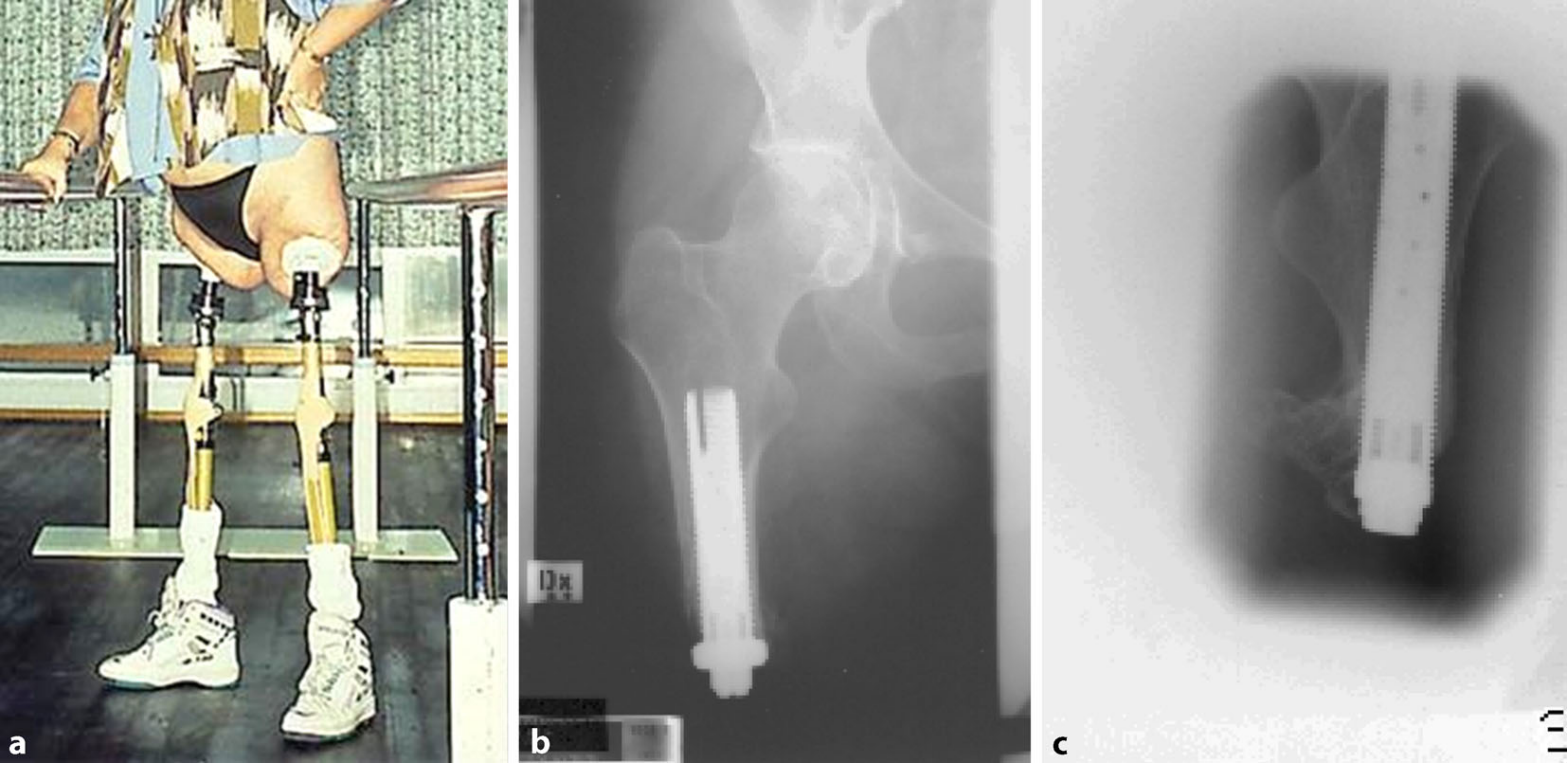
The first extremity osseointegration patient who was operated in 1990. The patient can stand up and walk with crutches (**a**). At that time, the implants were of modular design with a distal collar (**b** and **c**)

The first osseointegration treatment in an amputee was on 15 May 1990 on a 25-year-old woman, who had undergone bilateral transfemoral amputation at the age of 15 due to a tram accident. A titanium fixture was installed in her right residual femur. Six months later, a titanium abutment was connected to the well-osseointegrated implant (Fig. 1b). In 1991, a similar two-stage procedure was done on her left femoral stump (Fig. 1c). After postoperative rehabilitation, the patient could walk with crutches and exercise with cycling (Fig. 1a). The clinical trials were continued with a few transfemoral amputees as well as thumb amputees and a series of transradial amputees (TRAs) in the early 1990s [15]. These initial efforts provided valuable experience for the later standardization of the OPRA program.

## Improvements during the early 1990s

The initial clinical trials of osseointegration for amputees were more or less an extended application of the dental implants to the extremities. However, unlike the relative stable situation for oral and craniofacial applications, the implant system in extremities were under higher and unknown and unpredictable stresses during movements and falls. On the other hand, the muscle contractions and relaxations in daily life made the edge of the skin opening under frequent traction and twisting stresses against the percutaneous abutments. Therefore, although there were apparent functional improvements, there were inflammation/infection problems. In addition, mechanical complications due to overload led to fractures of the abutment screws, abutments, and fixtures.

The surgical techniques were also improved in the 1990s. At the first osseointegration operation, the importance of direct attachment of the dermal flap to bone was not well established. Experience from osseointegrated bone anchored hearing aids (BAHA) was adapted [16], which emphasized strict removal of all hair follicles in the skin in the 15 mm radius from the abutment opening and adequate soft tissue reduction at the end of the stump. This technique effectively reduced the risk of soft tissue problems by limiting soft tissue movement.

The early clinical trials also indicated that bone resorption could be problematic for long-term maintenance of the osseointegrated system. When placing the fixture flush with the distal end of the bone, resorption of the distal cortical bone was observed in some instances, which caused exposure of the threads of the fixture, and mobile soft tissues riding over the exposed threads led to inflammation. Therefore, a central position of the implant in the medullary canal and 20 mm embedment of the fixture end into the distal bone stump was later regarded as an optimal depth for fixture insertion.

## Initiation of the OPRA program

Based on experience from early clinical trials, R. Brånemark decided to standardize the implant system, surgical technique, and postoperative rehabilitation protocol. This programmed approach was named OPRA (Osseointegrated Prostheses for the Rehabilitation of Amputees). Standardization started with femoral implants (1998), and was followed by forearm (2003), humeral (2003), and thumb systems (2005). To date, the OPRA program provides standard operation procedures and rehabilitation protocols for femur, humerus, forearm, and thumb amputees.

## The standard implant designs

**Fig. 2:**
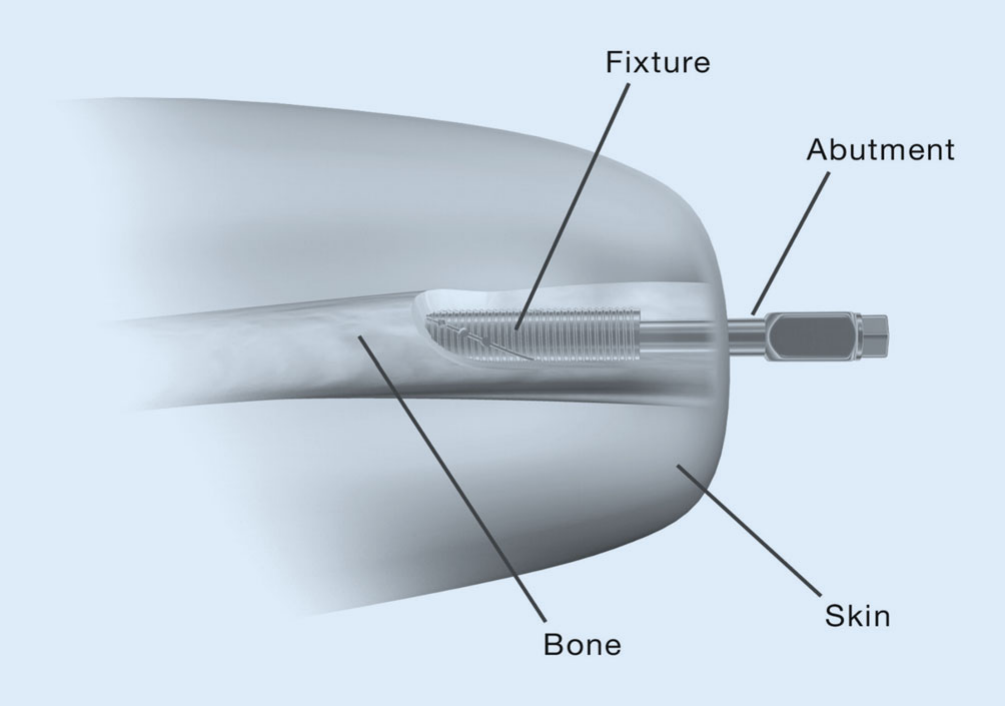
The basic implant design of the OPRA implant system. Three major components, the fixture, the abutment, and the abutment screw are used

The major components are the fixture, abutment, and abutment screw. The fixture has external threads for engagement with the inner surface of the bone cortex. The distal part of the fixture contains an internal fitting which connects the skin-penetrating abutment by press-fitting secured by an abutment screw (Fig. 2).

## Standard surgical technique

According to the OPRA protocol, the osseointegration operations are performed with two-stage surgeries. We have experienced that single-stage surgery could be done in selected patient with optimal bone quality and good compliance; however, in order to make a comparable analysis of all the advert events, the two-stage surgery with a fixed healing period is used in OPRA program. The 6‑month healing period is considered as long enough for the most undesirable bone situation and nonoptimal primary stability. At the stage 1 (S1) surgery, the fixture is inserted intramedullary into the bone stump. Close contact of the fixture threads to the inner cortex is necessary and good primary rotational stability usually indicates good future osseointegration. If the residual bone is too short or the distal bone end has unsatisfied quality due to disuse osteopenia, a cylindrical bone graft can be harvested from the iliac crest and transplanted to the bone end with compression of a graft screw. Cancellous bone graft from the iliac crest is often added to assure adequate distal bone closure. The bone grafts are compacted densely with a special instrument and maintained by healing components.

During the healing period, the stumps remain unloaded, but patients are allowed to use their socket prosthesis. At the stage 2 surgery (S2), the muscle endings are sutured to the periosteum 5–10 mm proximal to the bone end. The subcutaneous fat is removed at least 3 cm from the skin opening to guarantee a thin, hair follicular-free, and immobile skin around the abutment. The direct healing of skin to bone without any mobile soft tissue interface is crucial to reduce future soft tissue problems. The abutment is then inserted through the skin to the press-fit part of the fixture with compression applied by the abutment screw.

## Postoperative rehabilitation protocols

### Transfemoral amputees

**Fig. 3:**
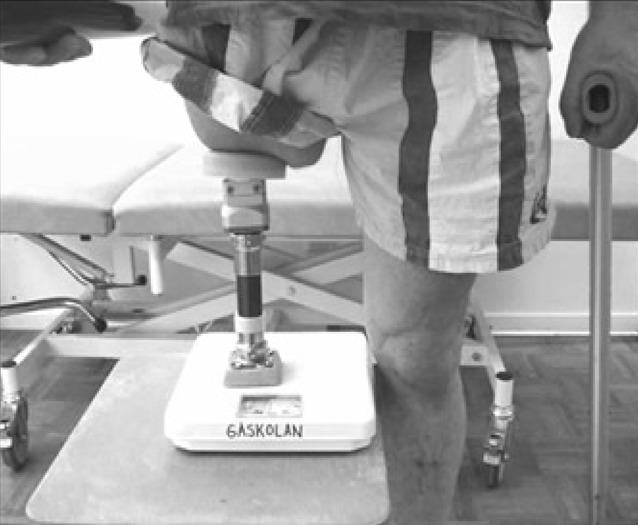
Transfemoral patient with short prosthesis for axial loading

The postoperative rehabilitation for transfemoral amputees starts about 2 weeks after S2 surgery by performing gentle exercises (i. e., range of motion [ROM] exercises without full voluntary muscle contraction). At 4–6 weeks after S2, when the skin penetration area and soft tissue are adequately healed, more active training begins. Initial training includes axial weight-bearing and weight-shifting standing on a short training prosthesis. The patient can measure the amount of weight put on the short training prosthesis using a normal bathroom scale (Fig. 3). In addition, the patient is given a general exercise program emphasizing more active training of hip ROM and muscle strength. The aim of the general exercise program is to stimulate bone strength by loading the bone–implant unit in additional directions other than axial.

**Fig. 4:**
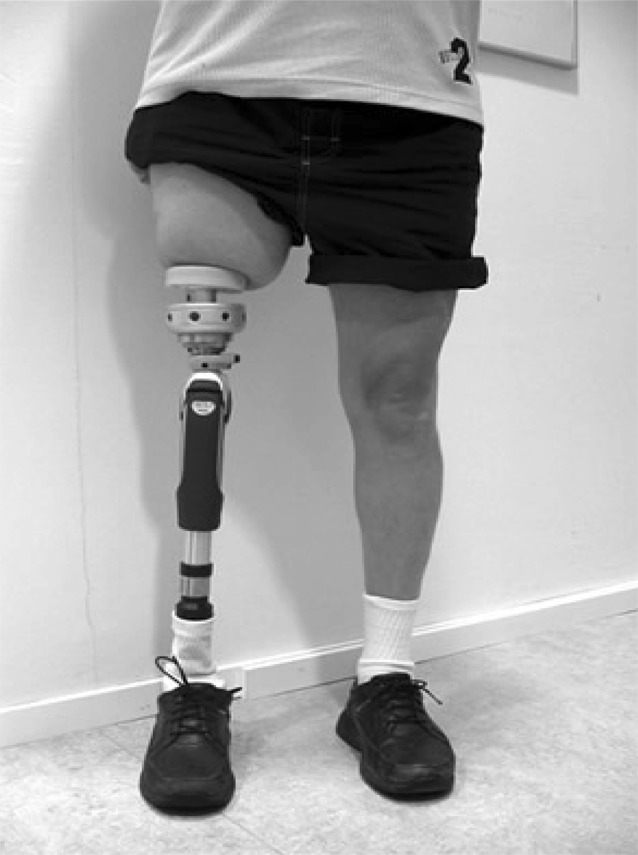
Transfemoral patient with long prosthesis

For patients with good bone quality and optimal primary stability, bearing on the short training prosthesis starts at 20 kg and is performed twice a day for 30min (Fig. 3). The patient is instructed to increase weight bearing by 10 kg each week until weight shifting to full body weight is achieved painlessly. Most patients report some pain during weight-bearing training, and pain recorded at VAS level 2 to 3 is considered safe. However, pain reported above VAS 5 should be avoided and weight-bearing exercises should be decreased to a more pain-free level. For all patients, the protocol includes 5–6 weeks of training with the short training prosthesis before prosthetic gait training on the definitive prosthesis starts (Fig. 4). Thus, prosthetic gait training starts at about 12 weeks after S2. During the first 2 prosthesis training weeks, the patient is instructed to use the prosthesis a maximum of 2 h/day, only indoors, and with the support of two crutches for limited weight-bearing on the prosthetic foot. The prosthesis wearing time, as well as prosthetic activity and weight-bearing, are gradually increased in the following weeks. The patient achieves full-day prosthetic use after 4–6 weeks. During the first 3 months of prosthetic use, walking should be done with double support (crutches or sticks). Based on X‑rays and the clinical status 6 months after S2, a decision is made by the team on walking without walking aid support both indoors and outdoors. Again, pain reported above VAS 5 should be avoided, and individual protocol progress should be slowed so as not to risk overloading the ongoing integration of the bone–fixture interface. To summarize, patients following the Normal Speed Protocol are treated for about 6 months after S2 operation. For patients with poorer skeletal conditions or inadequate primary stability, an individually designed, prolonged training protocol will be used which can take more than 12 months after the S2 surgery.

### Upper limb amputees

The prosthetic procedure after S2 surgery in the upper extremity depends on the amputation level [24]. The aim is to gradually increase the load on the implant over time. Specially developed osseointegration attachment devices, including the puck system, help to guarantee stable, reliable fixation of the prosthesis to the abutment. For transhumeral and transradial levels, the attachment device is available in two standard sizes, includes a quick-locking mechanism, has low weight, and is easy to keep clean. For thumb levels, the attachment device is made in one size and secured to the abutment using an Allen key. Some amputation levels require components to protect the implant from overload in rotation/torsion. A shock absorber to avoid unwanted shock peaks or forces and a temperature insulator can be built in if needed. In cases of myoelectric control, the electrodes can be held in place with flexible bars. The prosthetic cosmetic cover can meet up in contact with the distal tissue of the residual limb. All constructions that leave a hollow space and closed chamber in the penetration area have to be ventilated. Moisture can cause undesirable skin conditions and increase the risk of infection. A distal cap placed over the exposed abutment can be used for protection when the prosthesis is not worn.

### Transhumeral level amputations

**Fig. 5:**
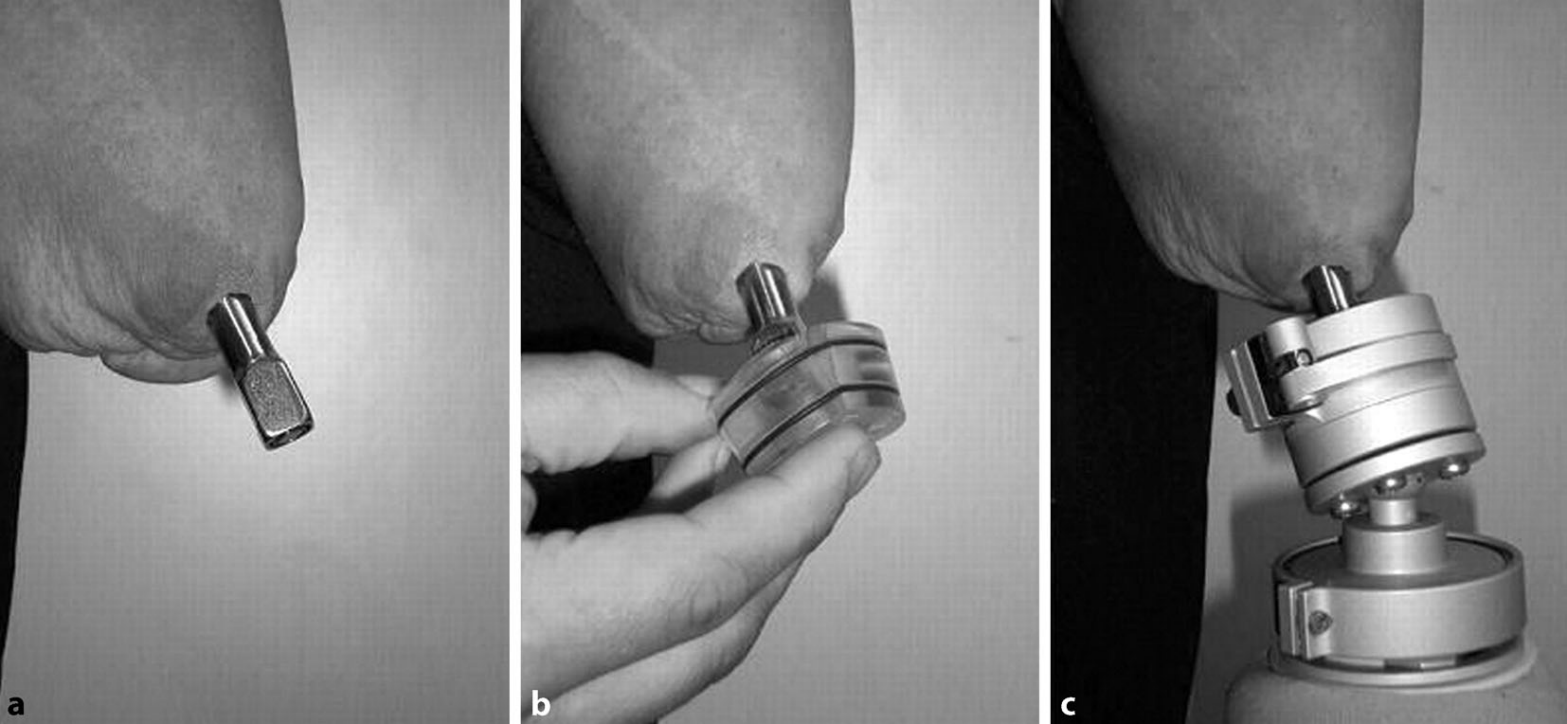
Puck system for transhumeral patient, consisting of standard attachment device (**a**), “puck” (**b**), and alignment components and rotation safety devices (**c**)

In transhumeral (TH) level amputations, it is important that the patient has maximum range of motion and good muscle strength before surgery to benefit the most from the increased motion produced by osseointegration. With a bone-anchored prosthesis, the joint closest to the prosthesis is loaded in an almost normal way, a situation the patient has not experienced since becoming an amputee. After S1 surgery, the patient is instructed to perform limited range of motion of the shoulder without pain. Three weeks after surgery, the patient can start to practice internal/external rotation of the shoulder to avoid rotational forces of the distal soft tissues. The aim is full range of motion by 6 weeks after surgery. Strengthening exercises for arms, shoulders, chest, and back muscles can also be started. After S2 surgery, the patient performs the same exercises as after S1. The prosthetic procedure begins 3 weeks after S2 surgery. The patient is fitted with a special training prosthesis which can be connect to increasing weights. During the first training week, 50–100 g are applied to the training prosthesis and these are increased each week (50–100 g) until the patient reaches the weight of the final prosthesis. Loading of the implant system is dependent on bone quality and pain. No pain above level 4, based on the visual analogue scale, is allowed. The patient should also perform axial weight loading twice daily by pressing the short training prosthesis against a bathroom scale according to a special treatment plan. The final prosthesis is fixed to the abutment by a standard attachment device, including spacers, alignment components, and rotation safety devices (Fig. 5). Usually 12 weeks after surgery, the patient can be fitted with a full-length prosthesis without grip function. Gentle exercises are performed with the prosthesis and these can be increased in intensity over time. Light bilateral activities can be performed. The patient can be fitted with a heavier functional prosthesis when the surgeon decides this is possible. Functional prosthetic grip training with bilateral activities can then be started.

### Transradial level amputees

**Fig. 6:**
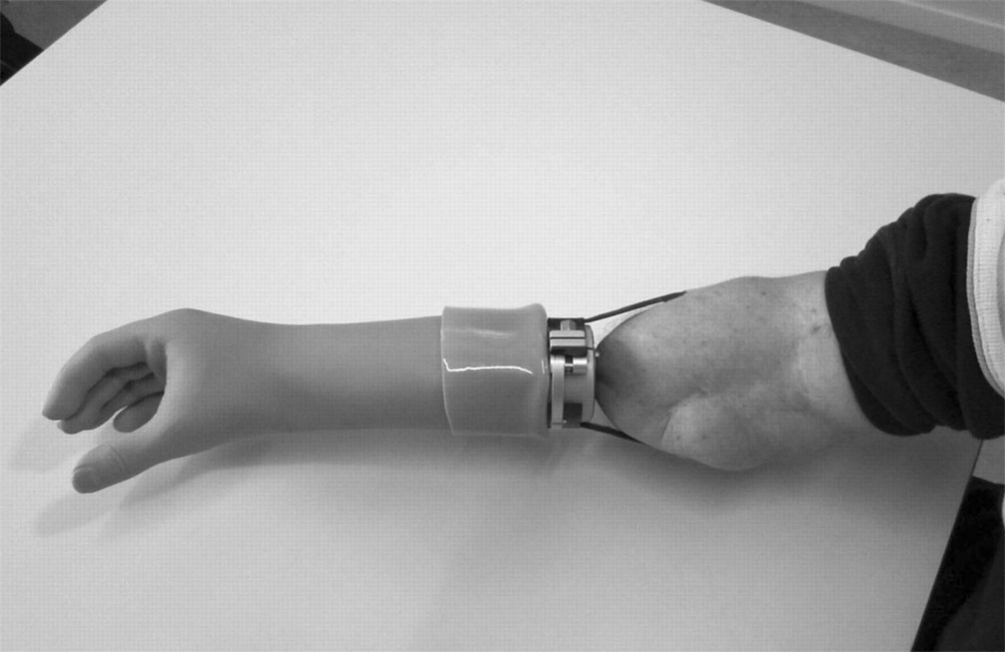
Transradial patient using an osseointegration myoelectrical prosthesis

In transradial (TR) level amputations, implants are normally placed in both the radius and ulna to obtain stable, reliable fixation. The double abutment status forms a uniquely individualized geometrical configuration, which makes the prosthetic connection special from amputations at other levels. An individual impression is crucial for successful prosthesis usage. The impression can normally be made 3 weeks after S2 surgery, using a special impression jig in which the abutment situation is captured in an optimal position. The impression jig allows the prosthetist to optimize the prosthetic alignment and check the prosthetic length. If there are plans for myoelectric control, the electrode sites are positioned using the jig as a reference. The geometrical situation of the abutment is copied into a plastic “puck”. The prosthesis can then be produced ready for delivery without any further control or checkpoints. If myoelectric electrodes are used, electrode holders are mounted. For this amputation level, cosmetic, myoelectric, body-powered, and passive hook/working prostheses have been fitted (Fig. 6).

Rehabilitation follows the same program as for the TH level, but no short training prosthesis is needed. The patient can wear a cosmetic or a lightweight myoelectrically prepared prosthesis and start to use the prosthesis as a support in daily activities. Based on the skeleton quality (X-ray) and pain assessment, a heavier prosthesis can be used and a general rehabilitation regime followed.

### Thumb level amputees

**Fig. 7:**
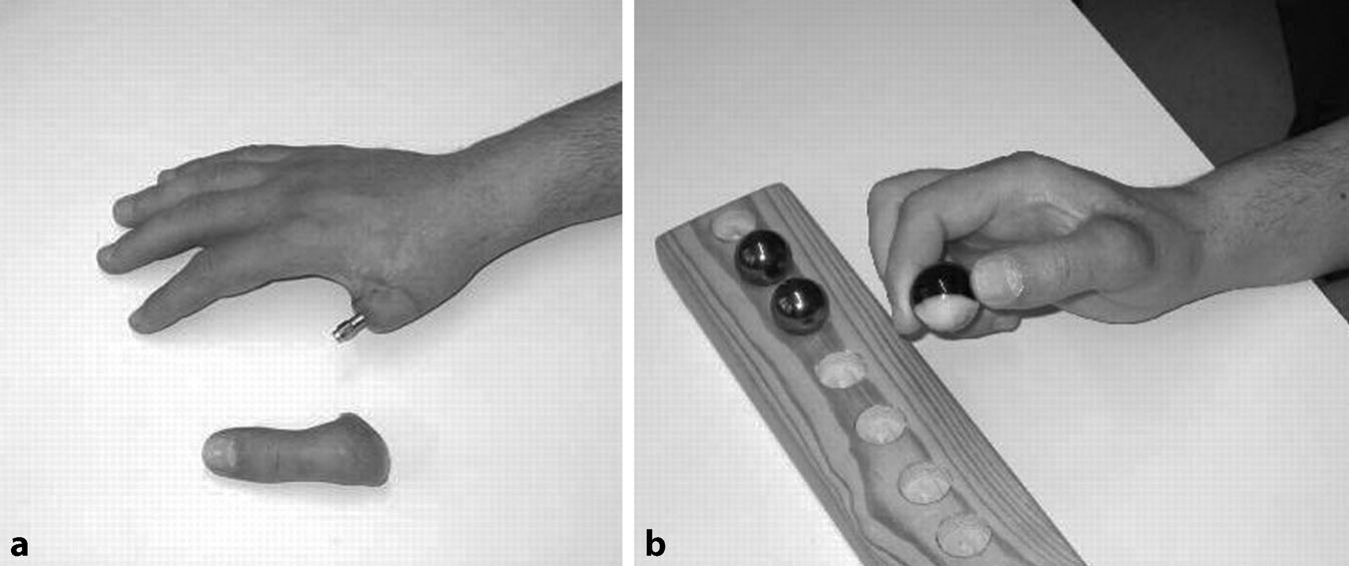
Thumb amputee (**a**) illustrating the use of an osseointegration prosthesis (**b**)

The prosthetic procedure starts with an impression to capture the shape of the residual limb in relation to the abutment position. This can be done if no edema exists, using silicone impression compounds. Some silicone prosthesis fabrication methods do not require this impression procedure. A special test prosthesis can be used to find the optimal prosthetic fingertip position. The final prosthesis is built around an attachment device with a hexagonal locking mechanism. A prosthetic inner frame, which gives the prosthesis stability, is connected to the attachment device. The outer part of the prosthesis is normally made of silicone and gives the prosthesis a high-definition aesthetic appearance (Fig. 7).

The healing time between the first and second operation at the thumb level was initially reduced to a 4-month interval; however, more recently several treatment have been performed successfully using a single-stage protocol. Range of motion has to be exercised postoperatively and edema must be reduced. The patient is fitted with a prosthesis when the edema has diminished. During the first 3 months after surgery, the thumb prosthesis should only be used for light activities of daily living. Heavy pinch/key grip should be avoided initially and the load increased over time. Pain should not exceed 4 on the visual analogue scale.

## Experience, long-term follow-up, and prospective studies

### Osseointegration for transradial amputees

Unlike lower limb amputees, transradial amputees (TRA) have no or minimal dysfunction on mobility or independence. Common socket suspension techniques for this level normally work well, even though the socket sometimes produces abrasion, tissue problems, discomfort, and most often reduced range of motion in the elbow joint. These problems are normally related to the length of the residual limb. A short residual limb is subjected to a high tissue load, while a long residual limb exposes more tissue to the socket interface. In addition, the current upper-limb prostheses do not restore the lost functions of forearms to the extent that lower limb prosthesis do for legs [17]. These factors made the patient inclusion very conservative and up to date only 11 patients were treated with osseointegration in Sweden. Ten men and one woman have been enrolled since 1990, including a bilateral amputee treated on both sides and two patients with dysmelia. Three patients received the standard OPRA treatment protocol, which was introduced in 2003. Fixture fractures occurred in 3 patients, all the fractured implants belong to the older design before the OPRA program started. The average stump length for patients with mechanical complications was shorter than patients without mechanical complications. We propose that the mechanical complications in TRAs are related to the special anatomy structures and kinematics of the forearm. In physiological conditions, the supination/pronation position during movement of the forearm is dependent on the delicate coordination of the pronator and the supinator muscle groups, the congruent movements of proximal and distal radioulnar joints, as well as the intact intraosseous membrane [18, 19]. In TRAs, the distal radioulnar joints, the distal part of intraosseous membrane, and the pronator quadrats muscle were excised. This nonphysiological condition makes the pronator teres (PT) muscle play a pivotal role in determining the position of the radius. The distal PT tendon inserts at the middle part of the radius shaft [20]. For amputations distal to the PT insertion, the deforming force generated by biceps brachialis (BB) and supinator (SN) is largely balanced by the PT and the radius is in a relatively parallel position to the ulna. At this position, the mechanical stress transmitted to the prosthetic connector is minimal. For amputations proximal to the PT insertion, the BB and SN provide an unopposed supination/external rotation deformity force to the radius. With application of the OI prosthesis, the force is transmitted to the ulna abutment by prosthetic connectors, resulting in torsion and bending stress to the implants. This hypothesis is supported by the fact that ulna fixture fracture were common and for TRAs with long radius stumps (residual radius ≥110 cm) no mechanical complication occurred. Strikingly, since the introduction of the OPRA program in 2003 no mechanical problem have occurred although two of the three patients had short stumps.

### Osseointegration for thumb amputees

From 1990–2014, a total of 13 patients with unilateral thumb amputations were treated with osseointegrated prostheses. There were 10 men and 3 women. Eleven had a traumatic amputation, and two were due to tumors. The OPRA program with a structured rehabilitation protocol and standardized implant components was introduced in 2005 and applied in 6 patients. The most common complications were mechanical failure necessitating change of components and superficial infection; 6 patients had no complications. Three patients had loosening, all in the early group. Seven patients (including all 6 after the introduction of the standardized protocol) had good osseoperception: grip strength (JAMAR) was 28.3 kg on the operated side vs 40.4 kg in the unaffected hand (70%), while key grip strength was 6 kg vs 9.1 kg, respectively. Hand function was 94% of the normal hand using the Sollerman test. The long-term follow-up indicates that osseointegration is a viable, safe procedure for thumb amputees to regain excellent function. The refined implant design and standardized protocol according to OPRA resulted in a 100% cumulative success rate with a 9‑ year follow-up so far.

### Osseointegration for transhumeral amputees

Between 1995 and 2010, 18 primary osseointegrated percutaneous implants and two implant revisions were performed in 18 transhumeral amputees; of those, 16 patients were available for follow-up at a minimum of 2 years (median 8 years; range 2–19 years). Two primary and one revised implant failed and were removed because of early loosening. A fourth implant was partially removed because of ipsilateral shoulder osteoarthritis and subsequent arthrodesis. The most common adverse event was superficial infection of the skin penetration site followed by skin reactions of the skin penetration site, incomplete fracture at the first surgery, defective bony canal at the second surgery, avascular skin flap necrosis, and one deep implant-associated infection. The implant system presented a survivorship of 83% at 5 years. Infectious complications related to the skin penetration site were easily managed with nonoperative treatment, which make it a potentially attractive alternative to conventional socket arm prostheses. This method was superior to socket prostheses, especially in transhumeral amputees with very short residual humerus in which the suspension of a conventional prosthesis is difficult.

### Prospective study in transfemoral amputees using the OPRA protocol

Between 1999 and 2007, 51 patients with 55 transfemoral amputations (TFA; 6 bilateral TFAs) were consecutively enrolled in a prospective, single-center, nonrandomized study and followed for 2 years. The OPRA protocol was strictly followed for each patient. All operations were performed at Sahlgrenska University Hospital, Gothenburg, Sweden and removal of the implant was regarded as the endpoint for failure. The main reasons for amputation were trauma and malignant tumor. The patients were reviewed at 3, 6, 12, and 24 months after the second-stage procedure. Any complications were recorded. Two validated, self-reported questionnaires, the Questionnaire for Persons with Transfemoral Amputation (Q-TFA) [11] and the Short-Form 36 Health Survey (SF-36) [21], were used to assess the functional outcome and health-related quality of life. Both were completed before the first-stage procedure and 12 and 24 months after the second.

Three patients withdrew from the study for reasons unrelated to the implant (one death from an unrelated cause, one severe dysfunction of the contralateral knee, and one lost to follow-up). Three patients had their implants removed during the study period due to inadequate osseointegration and one shortly after the study ended due to deep infection. The cumulative survival was, therefore, 92% at the 2‑year follow-up. At 24 months, 40 of 45 patients (89%) reported daily prosthesis use, compared with 57% (29 of 51) before the implant was inserted. One patient had severe pain and did not use the prosthesis at all, and 4 patients (2 patients with bilateral TFA) reported less than daily prosthesis use. The mean prosthetic use score improved from 47 (range 0–100) prior to the first stage to 79 (range 0–100) 2 years after the second stage procedure (*p* < 0.0001). All Q‑TFA scores improved (*p* < 0.0001), indicating improved prosthetic mobility, fewer problems, and an improved global situation. The overall situation as an amputee was stated to be improved in 69% of patients in the single question. The SF-36 physical function scores showed that general quality of life improved (*p* < 0.0001).

Superficial infection was the most frequent complication, occurring 41 times in 28 patients. On average there was, per patient, one superficial skin infection every second year. Most were treated effectively with oral antibiotics. Nine mechanical complications with the abutment and/or the abutment screw were reported in 4 patients, resulting in fracture or bending of the abutment and/or the abutment screw. All patients returned to normal function after the damaged components were replaced. There were no mechanical complications relating to the fixtures.

The cumulative success rate remains at 92% at the 5‑year follow-up (unpublished data).

### Osseointegrated human–machine gateway for long-term sensory feedback and motor control

For myoelectric arm prosthesis, electromyography (EMG) signal recorded by electrodes placed on the skin is limited to superficial muscles and susceptible to myoelectric interference, motion artifacts, and environmental conditions and, thus, considerably degrading the controllability of the prostheses. Compared with other percutaneous osseointegrated implant systems, a unique advantage of the OPRA implant design is that the central cannel inside the fixture and abutment makes the passage of electrodes possible. Together with researchers from Chalmers University of Technology in Gothenburg led by Max Ortiz Catalan, R. Brånemark developed a percutaneous osseointegrated (bone-anchored) interface which allowed for permanent and unlimited bidirectional communication with the human body. With this interface, an artificial limb can be permanently driven by implanted electrodes in the peripheral nerves and muscles of an amputee, outside of controlled environments and during activities of daily living, thus, reducing disability and improving quality of life.

In January 2013, a patient with an osseointegrated humeral prosthesis became the first amputee who received the osseointegrated human–machine gateway (OHMG) system for prosthesis control. The operation was performed without complications. To date, this special signal transduction system continually provides the patient with precise and reliable control of the prosthesis, regardless of limb position and environmental conditions, and with much less effort than surface electrode prosthesis. Furthermore, long-term stable myoelectric pattern recognition and appropriate sensory feedback elicited via neurostimulation was reported. The opportunity to permanently record and stimulate the neuromuscular system allows for the implementation of intuitive control and naturally perceived sensory feedback, as well as opportunities for the prediction of complex limb motions and better understanding of sensory perception. The permanent bidirectional interface based on OPRA implant design provide a critical step toward more natural limb replacement, by combining a stable attachment with permanent and reliable human–machine communication [22].

## Future perspectives

The standard implant system and treatment protocols of OPRA provide valuable information for evaluating the osseointegration method for amputee rehabilitation. Nowadays, the rigorous treatment regimen of OPRA protocol is undergoing further modifications. A second generation of implant system which provides even stronger mechanical endurance for long-term usage has just been applied clinically. The new implant system provides better primary stability and allows an accelerated rehabilitation process, such as one-step surgery and early weigh bearing. On the other hand, candidates with transhumeral amputations can be enrolled in a new clinical study using the OPRA human–machine gateway. Based on international collaborations, we are trying to combine the electrode implant with targeted muscle reinnervation (TMR) surgeries [23], and a more advanced prosthesis system to provide even better functional achievement for upper extremity amputees as well as in lower extremity amputees.

## Practical conclusion

- The direct attachment of osseointegrated prostheses avoids the inherent problems of socket suspension.
- Physiological weight bearing, improved range of motion in the proximal joint, as well as osseoperceptive sensory feedback enable better control of the artificial limbs by amputees.
- Pioneering efforts on extremity osseointegrated surgeries in Sweden and the development of the
- The Osseointegrated Prostheses for the Rehabilitation of Amputees (OPRA) program allows for structured rehabilitation with standard surgical techniques to achieve adequate bone–fixture integration.
- Long-term follow-up for femoral, transradial, transhumeral, and thumb amputee operations are positive.
- Patients with osseointegrated prostheses report an improved quality of life.
- Future possibilities for osseointegrated surgery include novel treatment options using electrode implantation.
